# General Medicine and Therapeutics

**Published:** 1894-08

**Authors:** 


### GENERAL MEDICINE AND THERAPEUTICS.

EDITED BY DR. LUTHER B. GRANDY.

### WHAT SHALL WE DO FOR OUR TYPHOID FEVER PATIENTS?

Dr. James Samson, in *Medical Age*, says:

Believing as I do, that quinine is always injurious to a typhoid patient, it is, I repeat, very important that enteric fever should never be treated as malarial disease—and the distinction, though sometimes difficult, can always or almost always be made pretty positively quite early in the case. The best possible treatment for malarial fever is about the worst possible for typhoid, and the best possible treatment for typhoid is absolutely useless in malaria. It is never necessary, I believe, to diagnose between typhoid and typho-malarial fevers. *There is no such disease as typho-malarial fever!*

Having assured myself that a case is indeed one of typhoid fever, I have for many years treated it with the least possible medicine, the least possible food, the least possible exertion, and the least possible excess of fever. I have divided my patients in a general way into two principal classes—one including young men and boys from sixteen to twenty-five years, and strong, robust young women of the same age, as those most likely to be of a serious type; and a second class including all others. In the first class I have found the symptoms grow more rapidly urgent than at other ages; in them the fever rises higher and sooner, and the wild delirium that means so much in them comes earlier. In this class especially is there apt to be that condition of the bowels that always forebodes trouble, and I have for a long time put all such cases on small doses of calomel, continued until the character of the evacuation is entirely changed, and from that time have been indifferent as to whether another single dose of medicine was administered. I have adopted the same treatment in all cases where there were dark or greenish or frequent stools, and never follow the calomel with a cathartic of any kind. I have put the patient to bed, to not again leave it for a minute till a week after the last vestige of fever, and have never yet lifted a single patient out of bed into a cold bath. I have tried to be always certain that the temperature should not exceed 102° or 102.5°, and I have doubted whether a temperature less than this needs to be much interfered with. I have during the past six years given not a single dose of antipyrin, antifebrin or phenacentin to any typhoid patient, and shall always feel that my own experience has been such as to contraindicate the use of these remedies in this particular disease at any stage or in any dose. I am sure their use has to be paid for in the days of convalescence, when a feeble heart has to make such a struggle. I have depended on cold sponging and cool surroundings entirely for my control of the temperature, and believe I have always found them sufficient. I have no desire to disapprove of the now famous Brand system. It has been marvelously successful when diligently used, but it is difficult of application in general practice, and interferes very much with the principles of constant quiet; for house to house treatment I shall not adopt it till more thoroughly convinced of its particular advantage, and until an easier mode of its application has been devised. To produce sleep I have depended chiefly on the cold sponge and have ceased using the bromides. I have not given the smallest dose of opium in any form, or chloral hydrate, in years, and feel now that I never expect to give them again. A few times I have resortod to sulphonal, and a very few times only. I have given all patients small doses of nitro-muriatic acid largely diluted—first because it seemed in the right direction, and again because it is perhaps well to give all patients something. I have not seen a case of tympanitic abdomen or persistent looseness of the bowels in many years, and have not had a case of intestinal hemorrhage since 1880; and these facts I attribute to absolute quiet and incessant vigilance in the matter of diet. A careful investigation has convinced me that a large majority of physicians recommend about twice the quantity of food I allow my patients, and I, of course, am inclined to insist that they all are wrong. So great a man as DaCosta gives three pints of milk and a pint of broth a day, and arrow-root at midday, and very many practitioners follow close on the same line. My own instructions are that the patient shall have milk, and milk only—three half pints a day, and never to exceed a quart. From this rule I never depart till a morning temperature has been normal; and to this, and this alone, do I attribute the remarkable exemption from bowel trouble and ulcerative disaster that I have experienced.

Perhaps the greatest triumphs in the management of severe forms of typhoid fever are those achieved toward the far off end of the disease, when a very feeble and rapidly failing heart, that may not even have been poisoned by antipyretics, warns us how close a place the patient has to pass through yet to escape. I need not repeat to you that it is now that stimulants, and nux vomica, and quiet, and no mistakes, can come so near performing a miracle. It is now that you can single-handedly fight away the grim monster if only the bowels are not harassing the victim. There are those practitioners, and there are very many of them, who give stimulants long before the heart fails; but I desire to express a strong doubt as to the wisdom of the method. The human system longs for alcohol as it does for nothing else when the proper time has come, and utilizes its every element; but I offer it as a demonstration of this law that whenever the physician can discover the odor of alcohol in the breath of his patient, no matter what the disease or what its stage, he may safely accept it as a reminder that the prescribing of it was a mistake.

I need not say that once a normal temperature has been touched nutritious fluids may be gently added to the patient's diet; nor need I say how long he should still lie absolutely quiet, except to add that I have never seen but a single case of relapse of the fever that was not certainly due to exertion or excess of food, and I have but little faith in calling any condition a true recurrence of the original disease.—*Columbus Medical Journal.*

---

### SPECIFICS FOR THE CURE OF INEBRIETY.

This was the title of a paper lately read by Dr. T. D. Crothers before the American Association for the Cure of Inebriety. He said:

The more accurately we study alcoholic and opium inebriety, the more impossible the assumption of specific remedies appears.

Inebriety is an insanity and often dependent on psychical and physical changes in the brain. A specific, to reach or even to neutralize any of these morbid changes, would require a degree of knowledge that at least a century of prognoses will hardly reach. There is no other subject upon which there is so little of scientifie and common sense reasoning. The assumption of specifics is a faith process, demanding acceptance from the mere statement involved in mystery, and appeals to the emotions. The moralist who asserts that the power of prayer and conversion is a true specific, places the theory of causes and remedy frankly and unreservedly before all.

Appeals to the testimony of reformed or cured inebriates for evidence of the value of the remedy is the same old delusion which for half a century has prevailed along the line of temperance work. Since 1845 hundreds of temperance revival waves have come and gone, and many thousands of enthusiastic honest victims have supposed they were permanently cured, only to find out their mistake and disappear. All these means and remedies used have been open and tangible. There has been no secrecy, simply an assumption of a certain chain of causes, to be met and cured by certain clearly defined remedies. The supposed cured men were equally as enthusiastic and positive as those who are now vaunting the secret gold cure specifics.

Another view of these gold cure specifics reveals the paradoxical position of claiming disease, and its curability in a brief time, by any remedy, secret or otherwise. The fact of disease makes the possibility of cure, by any chemical or other remedies, an impossibility in any brief period of a few weeks. A fractured bone is only healed by the slow operations of nature. The fracture and injury of the higher brain centers among inebriates must follow the same line of laws, and be governed by the same natural conditions.

The absurdity of such claims would be quickly recognized, even if the remedies were made known. But when the remedies are concealed, the subject becomes empiricism, unworthy of any serious attention. While it is unpleasant to note the credulity and dishonesty of the advocates of these specifics, it is cheering to realize that this is simply an epidemic stage in the evolution of this topic, which is a part of the natural history of every great truth. The disease of insanity has passed the same period. The disease of epilepsy is just emerging from its empiric stage. Even now the remedies to "cure fits" are the dying echoes of charlatanism that is passed. Gold cures, mind cures, cinchona cures, and every other cure for inebriety, that make claims as specifics, are simply beating up against the trade-winds of truth, depending on side currents of credulity, ignorance and delusion. Specifics for the cure of inebriety, *secret* or *open*, urged by any man or combination of men, are delusions and absurdities, opposed by all known principles of science. Inebriates, who are the new army of the insane, are not to be reached by specific remedies. Far above the levels of empiricism and quackery, they will be understood and treated in the near future.

---

### INFANTILE THERAPEUTICS.

Luzet (*British Medical Journal*) gives a critical review based on the work of Legendre and Broca. The special points really consist in the phases of the development in the infant, in the special feature of disease which here proceeds rapidly towards aggravation or recovery, and in the physiological peculiarities of more active metabolism, of more rapid absorption and circulation, of intact emunctories, and of a more impressionable nervous system. In regard to feeding, the regular increase in weight must be relied upon. A tuberculous nurse must not be employed, for if bacilli do not pass out with the milk, toxins can; in addition, the milk is less rich in fat and casein. Overfeeding the nurse must be avoided. Of course artificial feeding is only a method of necessity. The milk should be sterilized by means of steam under pressure. The therapeutical bath is used to reduce temperature. The bath is then gradually cooled down from 2° below the child's temperature to 30°; it is useful in enteric fever, severe scarlet fever, and cerebral rheumatism. The bath with increasing temperature is of value in collapse such as occurs in diarrhea. It may also be a vehicle for certain medicaments. More strictly therapeutical measure are then discussed in the following order: 1. Evacuating medication. The stomach tube is very useful, as well as intestinal injections and emetics. Apomorphine is dangerous. 2. Promotion of excretions.

The best diuretic is water. Large rectal injections of cold water constitute a good method of inducing diuresis. In uremia, icterus and all intoxications large injections are useful. Cold baths are also of service in increasing renal excretion. Digitalis is well borne by children. Diaphoresis is best obtained by physical agents, heat, wet sheet, hot drinks. Diuresis is more efficient than diaphoresis. 3. Sleep should never be interrupted in disease, with very few exceptions. It may at times be necessary to induce sleep. This may sometimes be done by removing something which interferes with sleep. Physical agents are again the best means, such as tepid baths, etc. Opium requires caution; chloral is useful. Bromides and antipyrin may be of service. 4. Fever is controlled by external agents, baths, etc. Quinine, antipyrin, sodium salicylate may be useful adjuvants. 5. Food is the best tonic. Alcohol is the best stimulant. 6. Antiseptic medication plays a very important part in infantile therapeutics. Carbolic acid in any form must be avoided. The mouth should be cleansed with alkaline lotions. Glycerine is a good non-fermentable medium. Antisepsis of the stomach may be procured by washing it out, and, together with the intestine, by the use of bismuth salicylate, salol, etc. Calomel is a powerful intestinal antiseptic. Antisepsis of the large intestine is obtained by means of irrigations containing naphthol, etc. It is indicated in typhlitis, appendicitis, membranous colitis, etc.—*Maryland Medical Journal.*

---

### The Dietetic Treatment of Phthisis.

Dr. Henry P. Loomis, of New York, lays down a few valuable rules upon the proper diet in phthisis, which may be of service. He states that all consumptives pass through three distinct stages in regard to their digestive powers: 1. The period during which digestion and appetite are unaffected. 2. The period from the time when gastric disturbance first begins until the stomach refuses solid food. During this time septic infection is more or less constant, the fever intermittent, and loss of flesh generally progressive. 3. The period from the time when solid food can no longer be taken without digestive disturbance until the death of the patient.

Some of the most important rules which should govern the dietetic treatment of phthisis may be formulated as follows: 1. Never take cough mixtures if they can possibly be avoided. 2. Food should be taken at least six times in twenty-four hours; light luncheons between the meals on retiring. 3. Never eat when suffering from bodily or mental fatigue or nervous excitement. 4. Take a nap or, at least, lie down for twenty minutes before the mid-day and evening meals. 5. Take only a small amount of fluid with the meals. 6. The starches and sugars should be avoided, as also all indigestible articles of diet. 7. As far as possible, each meal should consist of articles requiring about the same time to digest. 8. Only eat as much as can be easily and fully digested in the time allowed. 9. As long as possible, systematic exercise should be taken to favor assimilation and excretion; when this is impossible, massage or passive exercise should be undergone. 10. The food must be nicely prepared and daintily served; made inviting in every way. Dr. Loomis gives the following as a sample of a diet sheet in the early stages of phthisis:

*On awakening.*—Eight ounces of equal parts of hot milk and seltzer, taken slowly through half an hour.

*Breakfast.*—Oatmeal or cracked wheat, with a little sugar and an abundance of cream, rare steak, or loin chops with fat; soft-boiled or poached egg, cream toast, half-pint of milk, small cup of coffee.

*Lunch,* 10 A. M.—Half-pint of milk or small teacup of squeezed beef-juice with stale bread. 12, noon: Rest or sleep.

*Mid-day Meal,* 12:30.—Fish, boiled or stewed chicken, scraped meat-ball, stale bread and plenty of butter, baked apples and cream, two glasses of milk.

*Lunch,* 4 P. M.—Bottle kumyss, raw scraped-beef sandwich, or goblet of milk. 5:30 P. M.: Rest or sleep.

*Dinner,* 6 P. M.—Substantial meat or fish soup, rare roast beef or mutton, game, slice stale bread, spinach, cauliflower, fresh vegetables in season (sparingly).—*Medical Record.*

### INCOMPATIBLES.

Dr. James Kennedy, Professor of Pharmacy, University of Texas, Gaveston, says: "A mixture of antipyrin and nitrous ether will deposit green crystals if allowed to stand, providing there is not sufficient water present to keep the new substance in solution. Acetate of lead and sulphate of zinc are frequently ordered in combination for use in the treatment of inflammatory affections of the genital mucous membranes under the impression that the constringing power of these drugs is enhanced by so doing. This is a mistake. Double decomposition occurs when these two salts are dissolved together; sulphate of lead is precipitated as an inert substance, while the acetic acid is transferred to the zinc to form zinc acetate. Strychnine is sometimes prescribed in combination with the bromides of iodides. This is a dangerous practice, for the reason that after the mixture has been allowed to stand for some time the bromide or iodide of strychnine will crystallize and be deposited in the bottom of the vial, and as the last dose of the medicine is taken the greater portion of this powerful drug contained may be swallowed, and perhaps with fatal results. Illustrations of chemical incompatibility might be multiplied *ad infinitum.* The following rules should be borne in mind: Acids should never be prescribed in combination with alkalies or alkaline carbonates. Salts of the alkaline earths, *i. e.,* magnesium, calcium, barium and strontium should not be prescribed with soluble carbonates, tartrates or oxalates, and in the case of the three latter elements they should never be combined with soluble sulphates, phosphates, arsenates, phosphoric or sulphuric acid. The heavy metals, such as iron, manganese, lead, mercury, silver and zinc should never be prescribed in conjunction with the alkalies, their carbonates or oxalates. Silver should never be prescribed in combination with chlorides, bromides, iodides or hydrochloric acid or organic matter (substances of vegetable or animal origin). The soluble salts of lead should never be combined with chlorides, hydrochloric, hydrobromic, hydriodic or sulphuric acid."—*Medical Herald.*

### CROUP.

To summarize briefly the medical treatment of croup that will give the little victim the best chance of recovery is as follows: A warm room, the air of which is to be saturated with moisture; an emetic of turpeth mineral *to be repeated whenever the respiration becomes embarrassed;* if the bowels are constipated, a sufficient amount of mild chloride of mercury to move them thoroughly; ice to the throat, either in a rubber bag or bladder; if thirst exists, pellets of ice internally; quinine *to decided cinchonism, which is to be maintained;* a sufficient amount of hydrate of chloral to allay spasms of the air passages, with decided doses of belladonna; the inhalation of steam, medicated or not as may suit the peculiar belief of the individual practitioner; the juice of the pineapple, trypsin with carbonate of soda in warm water; to be supplemented by the administration of a suitable amount of easily digested food.—*Medical Brief.*

